# How do medical educators design a curriculum that facilitates student learning about professionalism?

**DOI:** 10.5116/ijme.5683.c2e0

**Published:** 2016-02-04

**Authors:** Vicki Langendyk, Glenn Mason, Shaoyu Wang

**Affiliations:** 1 Medical Education Unit, School of Medicine, Western Sydney University, Australia; 2 School of Community Health, Charles Sturt University, Australia

**Keywords:** Curriculum development, pedagogical theories, discourses of professionalism, design based research and curriculum reform

## Abstract

**Objectives:**

This study analyses the ways in which curriculum reform facilitated student learning about professionalism.

**Methods:**

Design-based research provided the structure for an iterative approach to curriculum change which we undertook over a 3 year period. The learning environment of the Personal and Professional Development Theme (PPD) was analysed through the sociocultural lens of Activity Theory. Lave and Wenger’s and Mezirow’s learning theories informed curriculum reform to support student development of a patient-centred and critically reflective professional identity. The renewed pedagogical outcomes were aligned with curriculum content, learning and teaching processes and assessment, and intense staff education was undertaken. We analysed qualitative data from tutor interviews and free-response student surveys to evaluate the impact of curriculum reform.

**Results:**

Students’ and tutors’ reflections on learning in PPD converged on two principle themes - ‘Developing a philosophy of medicine’ and ‘Becoming an ethical doctor’- which corresponded to the overarching PPD theme aims of communicative learning. Students and tutors emphasised the importance of the unique learning environment of PPD tutorials for nurturing personal development and the positive impact of the renewed assessment programme on learning.

**Conclusions:**

A theory-led approach to curriculum reform resulted in student engagement in the PPD curriculum and facilitated a change in student perspective about the epistemological foundation of medicine.

## Introduction

Leaving professional identity development to the enculturation process of clinical immersion is no longer an option in medical education due to the well documented detrimental effects of the hidden curriculum.^1^^-^^3^ However many clinicians remain committed to the traditional pedagogical model of role modelling and are sceptical of more formal educational methods.^4^^-^^6^ This distrust is not entirely unfounded, given the lack of consensus about how to structure the medical curriculum to support the development of medical professionalism.^6^^-^^8^

It is further exacerbated because professionalism curricula do not sit within the objective scientific paradigm which dominates the medical curriculum,^9^^,^^10^ and underpins students’ conception of what it means to be a medical professional.^11^ Given these tensions, professional development curricula can struggle for legitimacy in medical schools.^12^ This was a problem that like many others, we too experienced. How do we design a curriculum which will positively engage students so that they learn about and understand the relevance of medical professionalism?  In this paper we will demonstrate how an iterative, theory-led curriculum reform resulted in student engagement in the contested PPD curriculum and facilitated a change in perspective about the epistemological foundation of medicine.

### Our context

The Western Sydney University, School of Medicine (SoM) is a five year undergraduate programme, established in 2007. The first 2 preclinical years are founded on problem based learning and students spend one day a week in the hospitals gaining clinical experience. For the final 3 years of the course students are immersed in urban, rural and community clinical placements. Personal and Professional Development (PPD) is one of four themes that structure the curriculum. The PPD theme focuses on the social, cultural and ethical dimensions of medicine to facilitate the development of students’ professional identity. PPD is a recent curricular requirement for medical school accreditation, which has arisen in response to public concern about the medical profession’s increasing focus on technical expertise at the expense of a moral commitment to patient centred care.^12^

The SoM was established, rather expeditiously, as part of a comprehensive government response to significant patient safety concerns at the local hospitals in one of most disadvantaged areas of Sydney.^13^ Commencing with few staff and insufficient time to develop a home grown curriculum, the foundation curriculum was imported from an interstate medical school. During the inaugural years, student feedback about the PPD curriculum of the first 2 preclinical years was highly critical. PPD was denigrated as ‘soft’ and worse ‘useless.’ The negative discourse around PPD was noted by the national accreditation body for medical education during an early visit to the School, creating a strong impetus for curriculum reform.

### What is professionalism?

Curriculum development requires clarity about educational outcomes, the foundation on which content, instructional design, assessment and evaluation is established.^14^ The foundational curriculum lacked a clear conception of professionalism on which to base the PPD goals and outcomes. Developing that vision through scholarly inquiry and engagement with faculty members was a critical task.^15^^,^^16^

The traditional conception of professionalism as a list of individual traits and mutable attributes is underpinned by the assumption that identity formation is primarily an intrapersonal process.^17^ This discourse remains dominant in medical education with an accompanying pedagogical focus on the development of appropriate values and attitudes^16^ and the corresponding need to identify those students who exhibit problematic behaviour.^17^ However, an emphasis on individual responsibility can result in a punitive approach to professionalism, thereby missing the opportunity to develop a curriculum which focuses positively on the personal development of every student.^18^ Moreover, this discourse ignores the negative effects of the hidden curriculum^3^^,^^16^ which include the erosion of empathy^7^ and rise in cynicism in medical students.^19^

Medical education requires discourses of professionalism which explain the hidden curriculum, to enable students to identify and resist its negative impacts.^17^^,^^20^^,^^21^ The discourse of professionalism as an interpersonal process considers that identity is formed and enacted in relationships with others thereby illuminating the critical impact of hierarchical relationships on professionalism. Individual attributes remain important; however they are not fixed but mutable and co-created between people.^17^

The discourse of professionalism as a societal and institutional phenomenon, the most recent discourse to emerge in the literature, explains professional identity formation as a result of socialisation into a particular context.^17^ Professionalism cannot be understood apart from the social, cultural and political forces that operate within, and on, the healthcare system.^20^^-^^22^ Curriculum that intends to prepare students to participate in professional practice must move beyond the prevailing individual view to adopt a more complex construct of professionalism in which all three discourses operate and intersect in sometimes contradictory ways.^17^^, ^^20^^, ^^21^

### Pedagogical theories

This conclusion has implications for the pedagogical theories on which curriculum renewal and development should be based. Learning theories which focus on personal cognitive and behavioural processes are inadequate to guide curriculum founded on a conception of professional identity as a social process.^17^^,^^20^^-^^22^ Increasingly, educators and researchers have adopted sociocultural theories, such as Lave and Wenger’s Community of Practice (CoP), to illuminate the importance of socialisation for professional identity development.^23^ However, Lave and Wenger’s theory is insufficient to guide curriculum development because it reveals, but does not challenge the negative ramifications of the hidden curriculum.^24^^-^^26^

Lave and Wenger’s theory, based on the concept of CoP, explains learning as increased participation in the social and cultural practices of a community which consists of members who share a common activity and purpose.^27^^, ^^28^ Learners develop their professional identity through a process of socialisation into a new social language which involves learning to ‘walk the walk’ and ‘talk the talk’.^22^^, ^^29^^, ^^30^ Identity confirmation based on the need for social recognition is the motivating force that drives individuals to increase their participation in communities of which they are a member.^31^ Communities of practice explain how the hidden curriculum operates and reveals why the medical profession reproduces itself so successfully.^25^^, ^^26^

Lave and Wenger are philosophically committed to education as a mutual development process in which communities and learners influence each other; learning as a process of transformation not reproduction.^27^ However they do not clearly describe the processes which would enable novices to recognise or navigate problematic aspects of the dominant culture of communities of practice.^25^^,^^26^ Medical educators committed to transformative education face a dilemma; participation in communities of practice is critical for professional identity formation, but risks conformity to the dominating discourse. To navigate this paradox we draw on more radical pedagogy which aims for social change.

Mezirow’s theory of transformative learning aims to empower students to resist the negative effects of the hidden curriculum by promoting critical thinking.^32^ Mezirow argues that education should not only focus on instrumental learning, but must also challenge students to see the world from different perspectives which he calls, communicative learning.^32^ Communicative learning involves a critical questioning of the goals, values and norms of the communities of practice which are taken for granted. This type of learning requires engaging students in critical reflection, a process of questioning the validity of the premises of personal meaning perspectives which have been tacitly acquired, to reveal inaccurate assumptions that may be epistemic, sociocultural or psychic.^32^

Transformative learning is risky, and intellectually and emotionally challenging for teachers and students.^33^ Teachers require a philosophical commitment to the aims of transformative learning and an ability to engage students in exploring different ways of knowing.^32^^,^^34^ Their role is to create a safe and non-hierarchical learning environment to foster the development of peer dialogue and supportive relationships, critical for the success of transformative learning.^35^ 

Committed to curriculum development which aims to support students to develop a patient-centred professional identity, we utilised both Lave and Wenger’s and Mezirow’s learning theories to guide the redevelopment of the preclinical PPD curriculum. Lave and Wenger’s theory emphasises the importance of belonging and social recognition for professional identity formation; the positive and necessary components of enculturation.^36^ Medical education must aim to transform students into ‘the sort of person’ who does the kind of work that doctors do in the real world.^29^ Medical students need to develop a professional identity which necessitates them feeling that they are part of the profession.^29^^,^^37^ However, aiming to assuage the negative effects of enculturation, we also drew on Mezirow’s transformative learning theory to promote students to critically question the norms and values which shape the medical profession whose membership they seek.

## Methods

### Study design and methodology

We adopted design based research (DBR) methodology for our inquiry into theory led development of the PPD curriculum. DBR is an iterative approach to research embedded in practice; and encompasses phases of analysis and exploration, design and construction and evaluation and reflection.^38^^,^^39^ DBR shares the assumption common to both Lave and Wenger’s CoP and Mezirow’s transformative learning theories, that students cannot be understood separate from their learning context. The implication for educational research is that theory and practice cannot be considered separately; theory and practice are dialectically related. Therefore this methodology supports our dual and integrated aims of local practice improvement through action informed by theory and conversely, theory development through reflection on action in practice.^40^

Curriculum design and implementation is highly contextually dependant^41^ necessitating analysis which extends beyond the learner to encompass the sociocultural context. Therefore, we used Activity Theory (AT) which takes the whole complex learning environment as the unit of analysis, to guide our inquiry. Consistent with our research methodology and pedagogical approach to curriculum development, AT conceptualises learning, not as an accumulation of  discrete knowledge and skills but a process of transformation and identity construction as students develop their role/s in relation to the goals of a learning community.^42^

The founding principle of AT is the cultural mediation of individual action. Learning emerges from participation in collaborative purposeful activities, mediated by cultural tools, symbols and signs which fundamentally shape psychological development. This concept is represented by the AT triangle demonstrating the dialectic relationships between the subject (the learner), the object (the purpose of the activity) and the tools (mediators of learning) which may be either conceptual or technological.^43^

Acknowledging that the individual cannot be considered separately from the community of which they are a member, Engestrom extended the AT model to include the interacting and interdependent elements of community and the rules and division of labour which govern and mediate individual and collective action.^43^ Sites of contradictions and instability within and between activity systems represent historically accumulated structural tensions which can be targeted for educational interventions to facilitate learning.^43^

We used Activity Theory as a tool to identify and analyse the contradictions in the foundational PPD learning environment that were contributing to the negative discourse. The next version of the PPD curriculum was designed to address these structural tensions and to facilitate student learning towards the renewed curricular aims and objectives. After two years of implementation, another cycle of evaluation and analysis was performed to evaluate students’ attitudes to the reformed ‘soft’ professionalism component of the medical curriculum.

### Study participants

Ethical approval from the Human Research Ethics Committee of Western Sydney University was obtained for this study (research protocol, H9980). We invited all students from the first two years of the course and their PPD tutors to participate. All ten PPD tutors agreed to participate in a focus group discussion. From a cohort of 238 students across years 1 and 2, 212 students completed the surveys, a response rate of 89%. The average age of the year 1 and 2 cohorts was 21, and there was an even gender distribution in both years. 

### Data collection methods

During each of the two analytical cycles of the research project, multiple sources of data were obtained to populate the AT model and inform the analysis. For the first evaluative phase, the learning guides and materials, and curriculum and assessment documents were analysed, the results of routine quantitative university student evaluation surveys reviewed, and informal interviews with students and teachers undertaken. During the second evaluative phase data were obtained from a qualitative, free text response survey undertaken by students and focus group discussion with the tutors (refer to Table 1 for survey and focus group questions).

**Table 1:** Questions posed to students and tutors

Participants and data source	Questions
Student survey questions for free text responses	§ In what ways does the PPD curriculum contribute to your learning to be a health professional?
	§ How could the curriculum be improved?
	§ How do the PPD assessment activities contribute to your learning?
	§ What is the value of using the e-portfolio?
Tutor focus group stimulus questions	§ Do you feel that you have had any opportunities to contribute to the PPD curriculum, and if so, in what ways. And if you have, do you think the suggestions you have made have been adopted?
	§ Do you think that the worldview that the activities represent kind of correlate with your worldview on PPD and its role in the curriculum?
	§ What would you like to achieve as a PPD tutor. What are your main objectives?
	§ Looking back at your teaching what sorts of rules and guidelines do you have to follow?
	§ Do people feel part of the SOM?
	§ Why do you think the e-portfolio was introduced?

The surveys were completed anonymously and no identifying data were sought or collected. Two focus groups were conducted so that all tutors (n=10) could participate on at least one occasion. Two facilitators (GM and SW) used semi-structured^44^ open-ended questions to explore sites of tension in the PPD learning environment. The two facilitators had no academic responsibilities for the PPD theme but were members of the Medical Education Unit. The digital recordings were transcribed (by VL and GM) into written text for analysis.

### Data analysis

A thematic analysis was undertaken (VL and GM) of both the student and tutor qualitative data. The data from the year 1 and 2 student cohorts were initially analysed separately, however, because of the congruence of responses, the data were combined for final analysis. The data analysis was commenced individually and then developed into a collaborative process which involved comparing data, themes and more integrative analysis of meaning, back and forth, until shared understandings were achieved.^44^ The next step involved presenting and justifying the shared understandings to the third investigator (SW) which resulted in further, minor, refinements. Given that one of the authors (VL) is the academic co-ordinator of the PPD theme, reflections and dialogue about the potential for bias and attempts to minimise any effects, were a frequent and integral component of the research discussions.

The framework method^45^ was used throughout our interpretive practice to construct a set of matrices that allowed us to consolidate a view of the thematic elements and their association with the original interview and survey data. The adoption of this approach assisted in the promotion of a methodological and epistemic reflexivity^46^ because connections between the data and related themes could be explicitly articulated. The methodological transparency facilitated an ongoing awareness of the relationships between the data, the thematic elements emerging from the interpretation of the data and the aims and objectives of the research.

## Results

### Foundational curriculum analysis

Analysis of the foundational PPD curriculum revealed a contradiction between the espoused and the realised value of PPD in the medical curriculum. From an organisational perspective there was incongruity between the rhetoric of support for student professionalism (the rules and norms domain of AT), and the resources allocated to develop and promote the PPD theme. The curriculum theme lacked academic leadership: no individual had responsibility for curriculum evaluation and renewal, and tutor development (a community deficit). The PPD tutors were, and continue to be, casually employed and this represents a trend in the Australian higher education sector.^47^ This employment status, however, establishes a division of labour which did not empower tutors to instigate curriculum change in response to pedagogical problems.

There were further tensions within the foundational PPD curriculum structure. The learning goals and overarching aims were difficult to elucidate from curriculum documents and not clearly articulated to either students or teachers. Moreover, the curriculum was not well supported by scholarly resources, which possibly established the notion that PPD was just common sense. Addressing low level cognitive abilities of knowledge recall and superficial conceptual understanding, assessment reinforced this notion. The messages were clear; the School was uncertain about the outcomes of learning PPD. Students learn well from the hidden and the formal curriculum. PPD was ‘soft’ and ‘useless’.

### Curriculum reform

To address the sources of tension in the learning environment we focused on three overarching educational principles.

   •   Educational leadership is vital for staff development, curriculum development and advocacy of a contested curriculum.^48^^,^^49^

   •   Learning outcomes should target high level capabilities to achieve professional development and be clearly communicated to students and teachers.^50^^, ^^51^

   •   Alignment between learning objectives, curriculum content, assessment and pedagogical theory is needed to support students to achieve high level outcomes.^50^^-^^52^

Responding to the pressing need for curriculum reform and addressing the first principle, the School appointed a member of the Medical Education Unit to assume leadership of the PPD Theme (one of the authors, VL). The curriculum reform then commenced with an extensive process of education of, and consultation with, members of the School to renew the pedagogical outcomes for the PPD theme. Accordingly, the object of PPD learning has been reconceptualised in terms of personal growth and patient-centred professional identity formation^53^ which encompasses intra-personal, interpersonal and sociocultural dimensions. Targeting Tools, as mediators of learning in the AT model, the curriculum content, learning objectives and instructional design were changed to align with the renewed outcomes.

The curriculum content was revised to focus on key philosophical, moral, ethical, legal, socio-cultural and political issues relevant to the formation of a patient-centred professional identity.^54^ Topics include end of life care, the law and duty of care, ethical responsibilities of the medical profession to asylum seekers, gender and race in medical practice and research, anatomy and objectification of the body, and, stigma and mental illness. Each topic is supported by academic literature,^55^ resources from the humanities; film, television, poetry, art, literature, and/or media resources.^56^^-^^58^ Complex learning objectives addressing high level cognitive, affective behavioural and metacognitive domains were developed to demonstrate the connections between the curriculum content and learning processes to the overarching PPD aim of professional identity formation.

Principles from CoP and Transformational Learning Theory were used to guide instructional design. Didactic lectures and informal tutorials were replaced with structured small group collaborative learning facilitated by tutors with expertise and commitment to the biopsychosocial model of healthcare^59^ and students’ personal development. Both CoP and transformative learning theories advocate for a classroom structure that facilitates positive collaborative learning relationships between students and teacher/s to encourage student development. In the collaborative classroom students are encouraged to question and monitor each other’s’ work and to co-construct group goals, standards and values so that they take on increasing ownership of inquiry.^27^^, ^^33^

To promote enculturation into the medical profession, we utilised scenarios and developed tasks that would illuminate the cultural values and standards expected of healthcare professionals. However, we also presented ethical and moral dilemmas, alternative perspectives to the biomedical approach and social and political issues that required personal reflection and critical thinking and dialogue, to foster perspective change.^32^ Moreover, medicine was presented as a social, cultural, political and historical practice.^60^ We wanted students to appreciate that medical professionalism is dynamic and evolves. As the next generation of doctors, not only are they shaped by the profession, but the profession will also be shaped by them.

Facilitating transformative learning demands dedicated, highly skilled teachers who are able to tolerate ambiguity and encourage constructive dialogue on highly sensitive issues.^33^ To role model learning and personal change to their students, teachers must be reflexive about their teaching.^32^^,^^34^ They are also required to promote a transition of labour between themselves and students which requires a sensitivity to, and ability to manage, the dynamics of power within the group.^35^

Hence, staff selection and development was a high priority for PPD curriculum reform.^61^^,^^62^ Regular tutor meetings and workshops were scheduled to facilitate the formation of a PPD community of practice for support, collegiality and learning.^63^^,^^64^ The tutors were also provided with a handbook providing strategies for each tutorial topic to encourage critical reflection of students’ current meaning structures and worldviews and exploration of alternative perspectives.^65^

Potentially, assessment can undermine other attempts to move towards a more student centred pedagogy.^66^ Previously conducted as end of semester summative multiple choice or short answer questions, we changed the mode of assessment and moved to a progressive, formative approach to align with the developmental outcomes of the PPD Theme.^67^ A variety of individual and group based assessment formats were employed such as reflective journaling, research essays, class presentations and creative responses, and students were offered a choice of topics and assessment modes. A criterion-based, non-graded assessment system was adopted and students provided with opportunities for remediation and mentoring if they were not satisfactory in any assessment task.

The change towards a longitudinal and formative approach to assessment was not supported by the current technological learning management system (LMS) of the university. The LMS tool does not allow students to maintain a continuous record of their work and nor does it facilitate the provision of timely feedback as students work develops. Records of student learning are static finalised products to be uploaded for assessment, not dynamic documents which can be changed upon further reflection or in response to feedback provided by teachers and peers.

Our search for an alternative platform serendipitously coincided with the university’s decision to adopt, in trial mode, a new edition of an e-portfolio product. The e-portfolio platform contained functionalities which enabled submission of regular reflections on experience and previous work, and collaborations on developing work with peers and tutors, thereby promoting the type of formative educational experience that was aimed for. Introduction of new technology can be highly challenging for tutors,^68^ hence we provided them with intensive instruction. In turn, the tutors were expected to assist students during an introductory tutorial and provide ongoing support.

### Analysis of curriculum reform

Following two complete academic years of the renewed PPD curriculum a second evaluative phase was undertaken. Written qualitative feedback was sought from students about their learning in PPD and tutors discussed their experiences of facilitating student learning in focus groups. The student and tutor data were analysed separately. The themes which emerged from analysis of both data sets revealed a concordance which was unexpected given the different positions, roles and goals of tutors and students. We undertook a second level of analysis and compared and contrasted the data between the two groups. This process confirmed shared perceptions and common themes about learning and assessment in PPD and hence will be presented together (Table 2).

### Tutor collegiality

There was one theme however, that was specific to the tutors’ experience as staff members of the SoM, ‘Collegiality and Belonging’. Over the two years of working together to build and deliver the new curriculum the tutors reported the development of a strong sense of community and common purpose. As casual employees of the SoM, they considered that the PPD coordinator should have the major responsibility for curriculum development. However, because their contributions were sought, valued and employed in the curriculum reform, they felt a sense of ownership and pride in the renewed PPD curriculum.

“…it’s been good for my own personal growth and learning- my development too because when I teach I learn too. When I come up with ideas which I share here and that’s been valued and encourages me to come and contribute the way I have been and  I definitely feel part of the SoM” (Tutor)

They also reported a strong sense of belonging to the SoM which was important for staff involved in the delivery of a contested part of the curriculum. Moreover, even though tutors felt that they still had to “sell” the value of PPD to students, this had become a much easier task with the new curriculum. Increased student engagement combined with their own greater involvement with the SoM worked to legitimise their practice as teachers of PPD and increase their sense of achievement.

“…As a participant in the provision of PPD experiences, it is a course that I feel immensely proud of” (Tutor)

**Table 2:** Themes from qualitative analysis

Theme	Description
Becoming an ethical doctor	Identity development
	Moral, ethical and emotional development
	Holistic personal growth
	Professional behaviour
Developing a philosophy of medicine	Medicine as a social, historical, cultural and political practice
	Beyond the biomedical model
	The importance of the patient experience
Learning through discussion, critical thinking and reflection in a safe environment	Creating a safe open learning environment for students to learn
	Learning is about engagement and tailoring teaching to student needs
	Engendering reflection
	Being challenged and critical thinking
Assessment promotes learning	Assessment promotes learning
	Ungraded, progressive assessment facilitates personal development
	Diversity of assessment modes encourage personal exploration and expression
Electronic portfolio as a distraction to teaching and learning	Complexity of e-portfolio subsumed learning time
	Recognition of affordances but e-portfolio did not add value to learning
	E-portfolio as an inadequate tool for collaboration

### What students learned 

Ninety-five percent of participating students said that PPD contributed positively to their learning and professional development, however this may be an overestimate, given the 11% non-participation rate in the study. Students’ reflections about the way in which PPD contributes to their development as a health care professional converged on two principal themes ‘Developing a philosophy of medicine’ and ‘Becoming an ethical doctor’. The student themes corresponded with the tutors aims for student learning and with the overarching aims of the PPD theme.

The theme ‘Developing a philosophy of medicine’, encompasses students’ burgeoning understanding that medicine was more than a science. Opportunities to focus on the patient’s experience of illness, (which included interviews with patients in the community and narrative writing from the patient’s perspective) helped students to cultivate a biopsychosocial understanding of medicine.

“The PPD tutorials and assessments have given me a very good understanding of the social issues in medicine and the concept of 'patient-centred care'. I feel that PPD will make me a more aware medical student and doctor - allowing me to work with others to treat patients as a whole.” (Year 1 student)

“It introduces early other aspects of medicine, rather than purely science. It helps adaptation into the role of practitioner by setting clear expectations on ethics and professionalism which can be practiced in a clinical setting.” (Year 1 student)

Tutorial topics which addressed the socio-political dimensions of medicine promoted a broader perspective on the roles of the doctor. Second year students are required to write a group position paper about the health and wellbeing of refugee and asylum seekers in Australia. Many students said this activity, and other similar learning experiences, gave them a new understanding of the relationship between medicine and politics and the role of advocacy in professional practice.

“The PPD curriculum provides students with numerous topics related to current issues about medical systems, political and social problems which are essential to construct the fundamentals for a health professional.” (Year 2 student)

“PPD helps me develop into someone who has a better understanding of the broader, at times, controversial issues that surround the medical profession. It allows me to transcend the barriers of science into the clinical realm to better understand and connect with my future patients.”  (Year 1 student)

The theme, ‘Becoming an ethical doctor,’ -focused on students’ and tutors’ perceptions of how PPD assisted the development of a professional identity. PPD provided opportunities to explore the qualities important for ethical and patient-centred practice and imagine professional identities which encompassed more than technical competence. PPD also guided, and stimulated reflection on, appropriate ethical behaviour in the clinical environment. Students appreciated reflecting on how they and others were currently behaving with patients and each other, and also envisaging how to respond in an ethical way as their clinical responsibilities developed. Becoming an ethical practitioner was becoming part of their lexicon for which PPD received credit.

“Always an opportunity for insight and reflection that is often neglected with university studies.” (Year 2 student)

“Provides guidance and helps us nurture the skills to develop morally and ethically for a vocation in medicine.” (Year 2 student)

“It allows us to contemplate who we are and sort out what sort of doctor we want to become.” (Year 1 student)

The themes which capture student learning in PPD illustrate that they were experiencing an epistemological shift. The ‘soft’ curriculum, previously dismissed as irrelevant to professional development, facilitated a change in perspective about the epistemological foundation of medicine, taking them beyond a technical rationalist model of medical practice. Together with the development of a philosophy of medicine, students imagined becoming a particular sort of doctor; preparations and beginnings of an identity change.

### How students learned

Students and tutors emphasised the importance of the unique learning environment of PPD tutorials for nurturing personal development. The shared theme, ‘Learning through discussion, critical thinking and reflection in a safe environment’ was highly representative of student and tutor reflections on the dynamics of the PPD learning environment. Expressing personal views, being challenged and engaging with alternative perspectives was particularly valued by students. Peer discussions were especially important for learning, but students also cited engagement with the scholarly literature and resources from the humanities as critical triggers for perspectival change.

“Reading and discussion during tutorials contribute to my understanding since many people's opinions are being shared and discussed. Informal environment encourage discussion.” (Year 2 student)

“PPD gives an opportunity for thorough in depth discussions about important issues in medical practice that are not easily openly discussed without a platform for it, like PPD. This promotes an awareness and sensitivity to what it means to assume the professional identity.” (Year 2 student)

“Multi-faceted learning that gives an insight from other perspectives, specially the patient’s which is of utmost importance as they are who we will be interacting with and it is for their benefit that we will work for.” (Year 2 student)

Voicing personal opinions, engaging in value-laden discussions and learning about oneself and others requires a safe, supportive and nonjudgmental space which PPD provides. Students recognised this culture as fundamental to the ethos of PPD and vital for their engagement and learning. Correspondingly, tutors considered the establishment of this trusting environment to be one of the most critical components of their teaching responsibilities.

“PPD is a place where people feel safe it’s re-contextualising their learning-it’s a catalyst that opens up honesty. It gives them a chance to reflect. If you had a debriefing in a … context the venom might come out. But in PPD it’s safe.” (Tutor)

### The paradox of assessment

**Figure 1:**
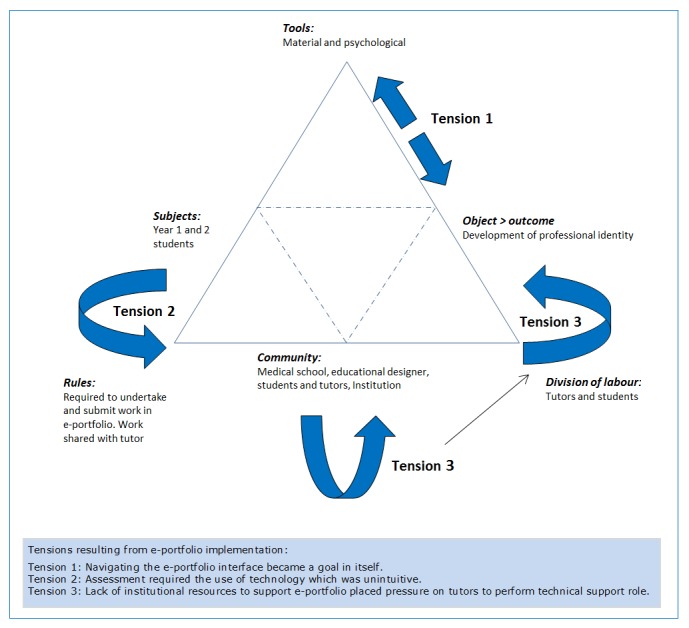
Activity theory and the e-portfolio

Students and tutors identified both positive (Assessment promotes learning) and negative aspects of the assessment reforms (The e-portfolio undermines learning). Given that the e-portfolio provided the technological and pedagogical structure for assessment, these two themes seemed contradictory. AT was used to analyse the tensions created by the introduction of the new technology and understand how both students and tutors could, notwithstanding the problems of the e-portfolio, claim that assessment promoted learning (Figure 1).

Technology must be intuitive and easy to use, even for the currently technological savvy generation of students,^69^ and institutional support is  critical for the successful implementation of educational designs that incorporate the use of new technology.^70^ Early after introduction of the e-portfolio it became apparent that the accompanying institutional technological support (community) was insufficient to address the significant technical issues (of the tool) that arose. The burden of helping the students with the new technology was consequently shifted on to tutors who, despite intensive training in the use of the e-portfolio, struggled to fulfil this role (tension 3). Both students and tutors found the interface of the e-portfolio technology complicated, unintuitive and difficult to use. Learning how to navigate the interface took up valuable tutorial time, disrupting rather than supporting PPD learning, leading to frustration, stress and feelings of ’time wasting’ (tension 1).

Despite instigating several strategies to increase support to students and tutors (workshops, online resources, one on one support), students and tutors continued to experience difficulty. In response, students proactively selected alternative technological tools to facilitate their group learning (such as Facebook and email). The assessment rules required collaborative engagement via the e-portfolio (tension 2). However, the relatively flat division of labour within the PPD learning environment allowed students to successfully negotiate the use of these alternate tools with the teaching and design staff, who responded by relaxing the rules to facilitate resolution of this tension in the learning environment.

Consequently students used the e-portfolio as a drop-box or repository for the products of learning rather than as a medium for the development of progressive knowledge construction and learning. In turn, tutors did not have access to the ideas that were being developed by students out of class time and were therefore not in a position to offer formative feedback through the e-portfolio as planned.

“The e-portfolio does not add anything to sharing information, collaborating ideas, and communicating. Students already have an efficient and much simpler means of doing so.” (Year 2 student)

“We don’t want it to end up about just using the e-portfolio and submitting and assessment. We want it still centred around their professional development. You don’t want the tutorial time eaten up by the logistics of using a system and all of that.” (Tutor)

If this pedagogical strategy for tying assessment to learning had failed, how could students and tutors claim that assessment promotes learning? Eighty-five percent of student respondents said that assessment enhanced their learning by encouraging a deeper exploration of topics discussed in class, stimulating personal reflection and providing both the choice to pursue topics of individual interest and opportunities for creative expression. Moreover, the progressive and more formative approach to assessment was appreciated, because it supported the developmental emphasis of the PPD theme. 

“The essay discussion based activities provide an open forum to express ideas about issues of particular interest to us, thereby creating a self-motivation to learn.” (Year 2 student)

“Solidifies own thoughts into a concrete piece of art/writing. This encouraged me to structure and organise my ideas discovering other truths along the way.” (Year 1 student)

Despite the contradictions introduced into the learning environment by the e-portfolio, interactions between the community, the tools and the division of labour, enabled students and tutors to maintain a focus on the object and outcomes for PPD. In a supportive and flexible learning community students were empowered to overcome obstacles to their learning by negotiating alternative pathways. Moreover, students and tutors reminded us that the most valuable learning was not mediated by the latest technological tool, but rather by discursive engagement with others during tutorials.

“…The profound benefits are the engagement in class – by, let’s say we are the students, sharing our learning ... getting consensus, affirming some of the things that we’ve reflected on, opening up new perspectives, that’s the benefit….. That’s just putting the cards on the table so you can play the game. And the game is in the tutorial.”  (Tutor)

Integrating feedback into the structure of the learning activity, rather than treating it as a separate item, enhances its value in the context of the formative development of students.

## Discussion

Professional development curricula struggle for legitimacy in medical schools and are contested on epistemic and pedagogical grounds.^12^ We conducted a theory-led curriculum reform which brought about a significant shift in the negative discourse around PPD amongst our students. PPD is now a valued part of the preclinical medical curriculum and, furthermore, no longer features problematically in curriculum committee discussions. This success can be attributed to a complex and iterative process of curriculum reform. The pedagogical outcomes were renewed and aligned with curriculum content, learning and teaching processes and assessment, and intense staff education was undertaken.

The importance of the development of a clear conception of the outcomes for a contested curriculum through scholarly inquiry and faculty engagement cannot be overstated.^16^^,^^21^ Curriculum which aims to contribute meaningfully to the development of a patient-centred professional identity must move beyond a narrow conception of professionalism as an intrapersonal process to include both intra-personal and sociocultural dimensions.^17^^,^^20^^,^^21^ Pedagogical theories that explain professional development as a sociocultural phenomenon are required to develop learning and teaching strategies that are in turn, based on this complex construct of professionalism. The content of this curriculum will necessarily be multi-disciplinary and focus on the interpretive rather than positivist epistemologies. Such a curriculum has to be supported by complex and integrated learning objectives which address cognitive, metacognitive, affective and behavioural domains.

The endorsement of PPD curricular aims by key academic leaders is essential for appropriate resource allocation to support and sustain curriculum reform.^53^ Moreover, this support is essential, though not sufficient, to bring on board the key stakeholders, the students. To sell a complex and contested curriculum to students it is also critical to be clear about its relevance and purpose, and remain ‘on message’ consistently throughout their learning and assessment experiences. Educational goals are clearly communicated to students when they are expressed through the learning and teaching activities and their assessment.^50^^-^^52^

Medical students will embrace a non-science curriculum when the educational goals are relevant to their professional development. The renewed PPD curriculum promoted an epistemological shift for our students; medicine is more than just a science. Furthermore, PPD not only influenced the way students thought about medicine; it also helped them to imagine the doctor they wanted to become. These ambitious and complex learning outcomes attest to effective educational alignment.

Curriculum development which aims to meet the challenge of engaging students in learning about professionalism cannot rely on an educator’s implicit knowledge but must be led by appropriate pedagogical theories. Students’ descriptions of how they were learning in PPD corresponded to those principles from Mezirow’s theory which guided educational design. Students found the curriculum topics provocative, authentic and highly relevant to their professional journey. Dedicated time in a safe learning environment to engage in critical questioning and dialogue with peers about challenging issues promoted learning and perspective change. Similarly, tutors’ conceptions of how students were learning in PPD resonated with Mezirow’s learning principles. Students engage with the ‘soft’ knowledge of PPD, when it is not presented didactically but as a discursive process of collaborative and rigorous inquiry with peers.

The PPD tutors, although highly experienced and committed, had struggled to engage students in the contested foundational PPD curriculum. To help tutors transition from implicit theories of teaching^71^ to teaching for transformation, staff development is critical.^65^ The formation of a collaborative team with a shared purpose and vision supports delivery of a challenging pedagogy. The concurrence between student and tutor conceptions of what was being learned, and how learning was happening in PPD, provides some evidence of the success of the formation of a CoP of PPD teachers.

Our research findings demonstrate the relevance of Mezirow’s theory^32^ as a foundation for curricula which aim to promote communicative learning, rather than instrumental learning. The focus of professionalism curricula, aiming to develop students’ professional values and attitudes, must be on communicative learning. The biomedical component of the medical curriculum has the easier task of facilitating instrumental learning. Our students reported that they learned about ‘becoming an ethical doctor’ and ‘developing a philosophy of medicine’ – these are communicative and not instrumental dimensions of learning. Furthermore, they attributed this learning to the theme of ‘….discussion, critical thinking and reflection in a safe environment’ and this aligns with key elements of Mezirow’s learning theory. Therefore, we would suggest that medical professionalism curricula require educators to utilise different learning theories than those that facilitate the instrumental learning required of the biomedical curriculum.

Cognisant of the potential for assessment to undermine communicative learning, we paid special attention to ensure that assessment aligned with the high level and complex goals of the curriculum. However the technological tool that was selected to facilitate the assessment programme proved problematic. As the curriculum developers, we sought solutions that would enable the e-portfolio to fulfil its theoretically sound, pedagogical promise of providing progressive feedback formative for learning. However, the students and tutors, free from theoretical commitments, negotiated their own practical solutions, which were effective. The students and tutors remained focused on the main goal of student learning. Identifying and resolving gaps between theory and practice requires close cooperation between curriculum developers, teachers and students. Theory and practice inform each other in a dialectical relationship which is integral to curriculum reform.

Given the potential for interactions and distributed effects of the changes that are made in any curriculum reform it is important to monitor the impacts and intervene when appropriate. The influence of the sociocultural context on the development of medical student professionalism cannot be overemphasised. Therefore the evaluation of formal curriculum addressing professional development requires a theory of analysis, such as Activity Theory, which illuminates the interacting and interdependent sociocultural elements that mediate individual and collective action.^43^ Curriculum alignment requires more than the synergy of educational goals, content, pedagogy and assessment. Medical educators also need to evaluate how the culture of the medical school and the educational institution can undermine or facilitate student learning.

### Limitations of study

An important limitation of this study was the omission of a formal systematic inquiry into the perspectives of the key academic staff of the School who were not directly involved in PPD teaching. We are, therefore, circumspect about drawing firm conclusions about the nature of the PPD discourse in the wider community of the School. We did however, have frequent meetings and discussions with the School academics, particularly during the process of negotiating the renewal of the PPD outcomes. Moreover, as members of the relevant committees, we can claim that PPD in the preclinical years is no longer a subject of concern as another accreditation visit looms. A further limitation to this study was the 11% rate of student non-participation. There may have been a disproportionate number of students amongst this group who did not value the curriculum reforms; hence we may have overestimated its positive impact. Furthermore, our results are highly contextual and relevance to other educational environments will need to be interpreted accordingly.

## Conclusions

Teaching for professional development in healthcare education is challenging worldwide.^12^ The methodological approach that we have undertaken to the reform of this contested component of the medical curriculum may provide guidance to healthcare educators facing similar challenges.

The most challenging obstacle to curriculum reform was the pervasive critique from students that PPD was ‘soft’ at best and at worst, ‘useless.’ However, medical education needs to engage students with the interpretive epistemologies to move them beyond a technical, scientific model of medical practice. We have completed the first two cycles of an iterative approach to the reform of a PPD curriculum which successfully engaged students in learning and generated a community of practice of teachers invested in the PPD theme. Addressing the tension caused by the e-portfolio, the next iteration of the PPD curriculum has been designed and implemented and will be analysed after a two year period. Curriculum reform is an ongoing process.

Although this study has investigated the PPD curriculum for the students during their pre-clinical years, this is not the most influential period for the development of students’ professional identity. Immersion in the clinical environment is the most important site for socialisation into the profession and where the complex effects of the hidden curriculum are profound.^1^^,^^2^^,^^72^ Therefore, we have also developed and implemented a PPD curriculum for students in their clinical years. This is a much more challenging pedagogical endeavour; however, student enthusiasm for PPD at least puts us in the game.

 “PPD is vital to our development - broadening scope from 'what' of sciences to the 'who' of a patient.” (Year 1 student) 

### Acknowledgments

The authors would like to acknowledge the contributions of all the PPD tutors and the medical students who took part in this study. We would also like to thank all our colleagues from the School of Medicine who either contributed to the curriculum development and administration of the PPD theme, or provided their support.

### Conflicts of Interest

One of the authors (VL) is the academic coordinator of the PPD theme and therefore may be predisposed to bias in terms of interpretation of students and tutors responses to the curriculum reforms. The other authors (GM and SW) are educators from the Medical Education Unit who have no academic responsibility for the PPD theme. We held weekly research meetings which involved frequent reflections on the relationship between our academic positions and perspectives and values as investigators and our relationships with the research participants and the interpretations and the stages of the research project. 

## References

[1] Coulehan J, Williams PC (2001). Vanquishing virtue: the impact of medical education.. Acad Med.

[2] Bloom SW (1989). The medical school as a social organization: the sources of resistance to change.. Med Educ.

[3] Newton BW, Barber L, Clardy J, Cleveland E, O'Sullivan P (2008). Is there hardening of the heart during medical school?. Acad Med.

[4] Christianson CE, McBride RB, Vari RC, Olson L, Wilson HD (2007). From traditional to patient-centered learning: curriculum change as an intervention for changing institutional culture and promoting professionalism in undergraduate medical education.. Acad Med.

[5] Shapiro J, Coulehan J, Wear D, Montello M (2009). Medical humanities and their discontents: definitions, critiques, and implications.. Acad Med.

[6] Haidet P, Dains JE, Paterniti DA, Hechtel L, Chang T, Tseng E, Rogers JC (2002). Medical student attitudes toward the doctor-patient relationship.. Med Educ.

[7] Hojat M, Vergare MJ, Maxwell K, Brainard G, Herrine SK, Isenberg GA, Veloski J, Gonnella JS (2009). The devil is in the third year: a longitudinal study of erosion of empathy in medical school.. Acad Med.

[8] Birden H, Glass N, Wilson I, Harrison M, Usherwood T, Nass D. Teaching professionalism in medical education: a Best Evidence Medical Education (BEME) systematic review. BEME Guide No. 25. Med Teach. 2013; 35(7):1252-66. 10.3109/0142159X.2013.789132 23829342

[9] Engel GL (1992). How much longer must medicine's science be bound by a seventeenth century world view?. Family Systems Medicine.

[10] Borrell-Carrio F, Suchman A, Epstein R. The Biopsychosocial Model 25 years later- principles, practice and scientific inquiry. Ann Fam Med. 2004; 2(6):576-582. 10.1370/afm.245 PMC1466742 15576544

[11] Waldstein SR, Neumann SA, Drossman DA, Novack DH. Teaching psychosomatic (biopsychosocial) medicine in United States medical schools: survey findings. Psychosom Med. 2001; 63(3):335-43. 10.1097/00006842-200105000-00001 11382261

[12] Cruess SR, Johnston S, Cruess RL (2002). Professionalism for medicine: opportunities and obligations.. Med J Aust.

[13] Walker B. Final report of the Special Commission of Inquiry into Campbelltown and Camden Hospitals (2004). NSW Government, Sydney, N.S.W. [Cited 30 December 2015]; Available from: http://trove.nla.gov.au/version/45513599.

[14] Biggs J (1996). Enhancing teaching through constructive alignment.. High Educ.

[15] Loeser H, O'Sullivan P, Irby DM (2007). Leadership lessons from curricular change at the University of California, San Francisco, School of Medicine.. Acad Med.

[16] Hafferty FW (1998). Beyond curriculum reform: confronting medicine's hidden curriculum.. Acad Med.

[17] Hodges BD, Ginsburg S, Cruess R, Cruess S, Delport R, Hafferty F, Ho MJ, Holmboe E, Holtman M, Ohbu S, Rees C, Ten Cate O, Tsugawa Y, Van Mook W, Wass V, Wilkinson T, Wade W (2011). Assessment of professionalism: recommendations from the Ottawa 2010 Conference.. Med Teach.

[18] O'Sullivan H, van Mook W, Fewtrell R, Wass V (2012). Integrating professionalism into the curriculum.. Med Teach.

[19] Testerman JK, Morton KR, Loo LK, Worthley JS, Lamberton HH (1996). The natural history of cynicism in physicians.. Acad Med.

[20] Hafferty FW, Levinson D (2008). Moving beyond nostalgia and motives: towards a complexity science view of medical professionalism.. Perspect Biol Med.

[21] Martimianakis MA, Maniate JM, Hodges BD (2009). Sociological interpretations of professionalism.. Med Educ.

[22] Swanwick T (2005). Informal learning in postgraduate medical education: from cognitivism to 'culturism'.. Med Educ.

[23] Amin A, Roberts J. Knowing in action: Beyond communities of practice. Research Policy. 2008; 37(2):353-69.

[24] Bathmaker AM, Avis J (2005). Becoming a lecturer in further education in England: the construction of professional identity and the role of communities of practice.. Journal of Education for Teaching: International Research and Pedagogy.

[25] Roberts J (2006). Limits to Communities of Practice.. J Management Studies.

[26] Hay K. Legitimate peripheral participation, instructionism, and constructivism: whose situation is it anyway. Educational Technology. 1993;33(3):33-8.

[27] Lave J, Wenger E. Situated learning: Legitimate peripheral participation. Cambridge, UK, Cambridge University Press;1991.

[28] Greeno JG (1998). The situativity of knowing, learning, and research.. American Psychologist.

[29] Gee JP (2000). Identity as an Analytic Lens for Research in Education.. Review of Research in Education.

[30] Lingard L, Reznick R, DeVito I, Espin S (2002). Forming professional identities on the health care team: discursive constructions of the 'other' in the operating room.. Med Educ.

[31] Paris S, Turner J. Situated motivation. In: Pintrich P, Brown D, Weinstein CE, editors. Student motivation, cognition and learning. New Jersey, Lawrence Erlbaum Associates Incorporated; 1994.

[32] Mezirow J. Fostering critical reflection in adulthood. A guide to transformative and emancipatory learning. San Francisco: Jossey- Bass; 1990.

[33] Taylor EW. The theory and practice of transformative learning: a critical review. Information Series No. 374.Columbus, ERIC; 1998.

[34] Cranton P. Understanding and promoting transformative learning: a guide for educators of adults. San Francisco, Jossey-Bass Higher and Adult Education Series; 1994.

[35] Berger JG (2004). Dancing on the Threshold of Meaning: Recognizing and Understanding the Growing Edge.. Journal of Transformative Education.

[36] Wenger E (2000). Communities of Practice and Social Learning Systems.. Organization.

[37] Goldie J (2012). The formation of professional identity in medical students: considerations for educators.. Med Teach.

[38] Barab S, Squire K (2004). Design-Based Research: Putting a Stake in the Ground.. Journal of the Learning Sciences.

[39] Anderson T, Shattuck J (2012). Design-Based Research: A Decade of Progress in Education Research?. Educational Researcher.

[40] McKenney S, Reeves TC. Conducting educational design research. New York, Routledge; 2012.

[41] Wong G, Greenhalgh T, Westhorp G, Pawson R (2012). Realist methods in medical education research: what are they and what can they contribute?. Med Educ.

[42] Blåka G, Filstad C (2007). How does a newcomer construct identity? A socio-cultural approach to workplace learning.. International Journal of Lifelong Education.

[43] Engeström Y. Expansive Learning at Work: Toward an activity theoretical reconceptualization. Journal of Education and Work. 2001; 14(1):133-56.

[44] Mason J. Semistructured interview. The SAGE encyclopedia of social science research methods. Thousand Oaks, CA: Sage Publications, Inc.; 2004.

[45] Gale NK, Heath G, Cameron E, Rashid S, Redwood S (2013). Using the framework method for the analysis of qualitative data in multi-disciplinary health research.. BMC Med Res Methodol.

[46] Johnson P, Duberley J. Reflexivity in Management Research*. J Management Studs. 2003; 40(5):1279-303.

[47] Kimber M (2003). The Tenured 'Core' and the Tenuous 'Periphery': The casualisation of academic work in Australian universities.. Journal of Higher Education Policy and Management.

[48] Elizondo-Montemayor L, Hernández-Escobar C, Ayala-Aguirre F, Aguilar GM. Building a sense of ownership to facilitate change: the new curriculum. Int J of Leadership in Educ. 2008; 11(1):83-10.

[49] Ramsden P (1998). Managing the Effective University.. Higher Education Research & Development.

[50] Meyers NM, Nulty DD (2009). How to use (five) curriculum design principles to align authentic learning environments, assessment, students' approaches to thinking and learning outcomes.. Assessment & Evaluation in Higher Education.

[51] Biggs JB. Tang, C. Teaching for quality learning at university: what the student does. Berkshire, McGraw-Hill Education (UK);2011.

[52] Prideaux D (2003). ABC of learning and teaching in medicine. Curriculum design.. BMJ.

[53] Irby DM, Cooke M, O'Brien BC (2010). Calls for reform of medical education by the Carnegie Foundation for the Advancement of Teaching: 1910 and 2010.. Acad Med.

[54] Braunack-Mayer AJ, Gillam LH, Vance EF, Gillett GR, Kerridge IH, McPhee J, Saul P, Smith DE, Wellsmore HM, Koczwara B, Rogers WA, McNeill PM, Newell CJ, Parker MH, Walton M, Whitehall JS (2001). An ethics core curriculum for Australasian medical Schools.. Med J Aust.

[55] King KP (2004). Both Sides Now: Examining Transformative Learning and Professional Development of Educators.. Innovative Higher Education.

[56] Salmon P, Young B (2011). Creativity in clinical communication: from communication skills to skilled communication.. Med Educ.

[57] Czarny MJ, Faden RR, Sugarman J (2010). Bioethics and professionalism in popular television medical dramas.. J Med Ethics.

[58] Weaver R, Wilson I, Langendyk V (2014). Medical professionalism on television: student perceptions and pedagogical implications.. Health (London).

[59] Engel GL. The need for a new medical model: a challenge for biomedicine. Science. 1977; 196(4286):129-36. 10.1126/science.847460 847460

[60] Starr P. The social transformation of American medicine. Cambridge, Basic Books; 1982.

[61] Steinert Y, Cruess RL, Cruess SR, Boudreau JD, Fuks A (2007). Faculty development as an instrument of change: a case study on teaching professionalism.. Acad Med.

[62] Eisen MJ (2001). Peer-Based Professional Development Viewed Through the Lens of Transformative Learning.. Holistic Nursing Practice.

[63] Gravett S (2004). Action research and transformative learning in teaching development.. Educational Action Research.

[64] Wenger E, McDermott RA, Snyder W. Cultivating communities of practice: a guide to managing knowledge. Boston, Harvard Business Press;2002.

[65] Taylor EW (2007). An update of transformative learning theory: a critical review of the empirical research (1999-2005).. International Journal of Lifelong Education.

[66] Fook CY, Sidhu GK. Authentic assessment and pedagogical strategies in higher education. Journal of Social Sciences. 2010;6(2):153-61.

[67] Yorke M. Formative assessment in higher education: moves towards theory and the enhancement of pedagogic practice. Higher Education. 2003;45(4):477-501.

[68] Schrum L (1999). Technology professional development for teachers.. ETR&D.

[69] Lei J. Digital natives as preservice teachers: what technology preparation is needed? Journal of Computing in Teacher Education. 2009;25(3):87-97.

[70] Kennedy G, Dalgarno B, Bennett S, Gray K, Waycott J, Judd T, et al. Educating the next generation - a handbook of findings for practice and policy. Strawberry Hills: Australian Learning and Teaching Council; 2009.

[71] Trowler P, Cooper A. Teaching and Learning Regimes: Implicit theories and recurrent practices in the enhancement of teaching and learning through educational development programmes. Higher Education Research & Development. 2002; 21(3):221-40.

[72] Newton BW, Barber L, Clardy J, Cleveland E, O'Sullivan P (2008). Is there hardening of the heart during medical school?. Acad Med.

